# A Systematic Review on Technology-Supported Interventions to Improve Old-Age Social Wellbeing: Loneliness, Social Isolation, and Connectedness

**DOI:** 10.1155/2020/2036842

**Published:** 2020-07-12

**Authors:** Francisco Ibarra, Marcos Baez, Luca Cernuzzi, Fabio Casati

**Affiliations:** ^1^Department of Information Engineering and Computer Science, University of Trento, Trento 38123, Italy; ^2^Department of Electronics and Informatics, Universidad Católica “Nuestra Señora de la Asunción”, Asunción, Tte. Lidio Cantaluppi y Guillermo Molinas, Paraguay; ^3^Tomsk Polytechnic University, Tomsk 634050, Russia

## Abstract

**Background:**

This review studies technology-supported interventions to help older adults, living in situations of reduced mobility, overcome loneliness, and social isolation. The focus is on long-distance interactions, investigating the (i) challenges addressed and strategies applied; (ii) technology used in interventions; and (iii) social interactions enabled.

**Methods:**

We conducted a search on Elsevier's Scopus database for related work published until January 2020, focusing on (i) intervention studies supported mainly by technology-mediated communication, (ii) aiming at supported virtual social interactions between people, and (iii) evaluating the impact of loneliness or social isolation.

**Results:**

Of the 1178 papers screened, 25 met the inclusion criteria. Computer and Internet training was the dominant strategy, allowing access to communication technologies, while in recent years, we see more studies aiming to provide simple, easy-to-use technology. The technology used was mostly off-the-shelf, with fewer solutions tailored to older adults. Social interactions targeted mainly friends and family, and most interventions focused on more than one group of people. *Discussion*. All interventions reported positive results, suggesting feasibility. However, more research is needed on the topic (especially randomized controlled trials), as evidenced by the low number of interventions found. We recommend more rigorous methods, addressing human factors and reporting technology usage in future research.

## 1. Introduction

Social interactions significantly impact the quality of life of adults in general and older adults in particular. Health risks have been associated with the characteristics of each individual's social network, such as small size [1, 2], lack of diversity [3], infrequent contacts [4], and perceived social isolation [5]. Limited or poor social relationships have been shown to increase the risk of dementia by 60 percent [6]. Loneliness is a known risk factor for depression [7] and has been associated with increased risk of death and with functional decline [8]. A meta-analytic review of 70 studies [9] has shown that the likelihood of mortality increased by roughly 30 percent for reported loneliness, social isolation, and living alone, an effect comparable to those of smoking and obesity.

Several studies report that both loneliness—a subjective measure referring to the “unpleasant” lack of (quality of) social relationships [10]—and social isolation—an objective measure referring to the lack (absence or low number) of social relationships [10]—increase as we age. Studies on different geographical and cultural regions report on varying levels of loneliness and social isolation depending on the country and the scale used. Older adults experiencing loneliness have been reported in the 20% to 30% range in Europe [11], 19% to 32% in the United States [12, 13], and 29.6% and above in China [14]. These figures give us an indication of the extent of the barriers and challenges to social participation in older adults [15].

Indeed, several risk factors such as sensory incapacity and reduced mobility, as well as reductions in the quality and frequency of contact and requirements for long-term care or additional support, are associated with loneliness in old age [16]. Predictors of loneliness and isolation for older adults include health problems (such as chronic illness and cognitive decline), widowhood, and living far from relatives or alone [17]. Even when older adults engage in a conversation, interactions can be challenging. Williams and Nussbaum [18] reported on the challenges of intergenerational conversations, such as patronizing speech, painful disclosures, and underutilization of topical resources. In particular, lack of conversation topics can generate anxiety in intergenerational conversations [19]. These factors might affect older adults, but, more importantly, they are usually beyond the affected person's control [20].

Technological innovations, along with social and economic changes, have made interconnected devices a commonplace, thus creating opportunities for interaction [21, 22]. Nonetheless, few reviews have focused on technology-supported interventions aiming at reducing loneliness and social isolation for older adults. Choi et al. [23] conducted a meta-analysis on computer and Internet training interventions, but did not cover newer devices. More recent reviews have analyzed assistive technologies and ICT interventions, but these have included interventions for general age-related problems, such as falls and medication management [24], or considered interventions that required colocated participation, such as playing video games [25].

In this systematic review, we focus instead on interventions enabling long-distance interactions through technology-mediated communication, targeting loneliness and social isolation in old age. Our objective is to identify the findings and limits of the knowledge acquired so far and to emphasize areas where further research is needed. More specifically, for the interventions analyzed, we investigate the following research questions:

 
*RQ1*. What challenges of long-distance interactions are addressed and how?
 
*RQ2*. Which technologies are used by interventions and how?
 
*RQ3*. What are the social interactions facilitated by interventions and with whom?

In the following, we discuss our investigation methods and results.

## 2. Methods

### 2.1. Search and Information Sources

We conducted a systematic review [26], reported here following the PRISMA statement guidelines [27], searching Elsevier's Scopus database for related work published in English until January, 2020. The search query was constructed using keywords for *older adults* (older adult OR older people OR senior OR elder OR ageing OR aging), the *target problems* (loneliness OR social isolation), *means for interaction* (technology OR Internet OR ICT OR IT OR computer OR tablet OR mobile OR smart phone), and *focus on social (communication* OR *social interaction* OR *social network* OR *social networking* OR *social participation* OR *social cognition* OR *community)*.

### 2.2. Eligibility Criteria

We analyzed the title and abstract of each of the 1178 search results and verified whether the publication targeted older adults. For the purpose of this review, we adapted the definition of older adults by the WHO [28] and considered eligible studies reporting on participants aged 65 and older, or with a mean participant age above 65 years of age. In addition, we considered eligible papers that conformed to the following criteria:

- The work included an intervention (i.e., action taken to improve a situation)
- The interactions with people were long-distance
- The intervention supported mainly technology-mediated communication
- The impact on loneliness or social isolation was evaluated

Despite our focus on loneliness and social isolation, we considered social connectedness, the experience of belonging and relatedness among people, as a valid outcome because it is related to the (dis)satisfaction with contact quantity and quality [29].

### 2.3. Study Selection

**Figure 1:**
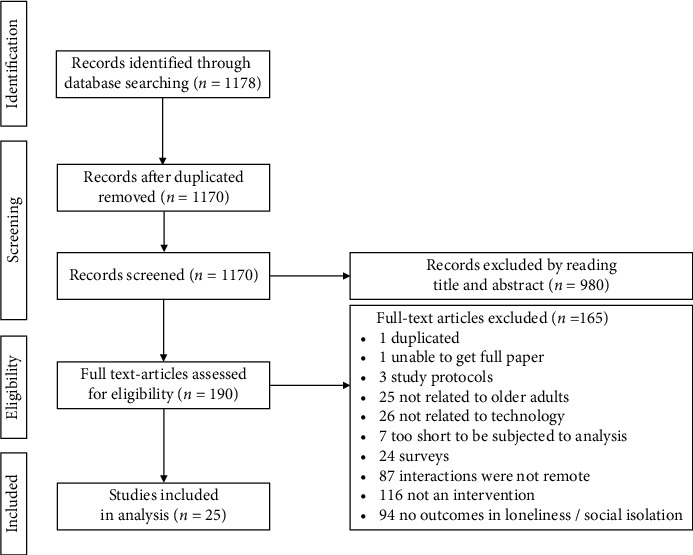
The full selection process, following the PRISMA statement guidelines.

After the identification and screening phases, 190 publications were left (see Figure 1). These publications were read fully and again discarded if not conforming to our inclusion criteria. An additional exclusion criterion for full paper screening was insufficient detail in reporting interventions, which would prevent a meaningful analysis. After a detailed inspection, 25 publications were left for full analysis.

### 2.4. Analysis and Synthesis

The review follows a narrative approach to the synthesis of results, given the heterogeneity of the studies included. In order to answer our research questions,

- On the challenges of long-distance interactions addressed by interventions (RQ1), we analyze intervention strategies and outcomes used to accomplish the study goals
- On the technology used to support interventions (RQ2), we account for the technology and devices, as well as the use of the technology by older adults
- On the social interactions enabled (RQ3), we describe the different contexts of interaction and the contacts reached by participants

Some authors were contacted to clarify the devices used in their interventions and the strategies that participants used to meet new people online. For two studies [30, 31], we were able to contact and get a reply from the authors.

## 3. Results

### 3.1. Study Characteristics and Outcomes

We start by summarizing the interventions analyzed. There were 13 interventions that considered loneliness as a primary outcome [30–42], one of which also considered social isolation [34]. Another 12 had loneliness and/or social connectedness as secondary outcomes (see Table 1, Outcomes).

Six interventions conducted qualitative studies, relying on direct or indirect (e.g., reports by staff) observation, questionnaires, and interviews (see Table 1, Study methods). All qualitative studies had positive outcomes, reporting mainly a decrease in loneliness [30, 32, 40, 43] (see Table 1, Conclusion). We must note, however, that some interventions described their results as “perceptions” or “being anecdotal” [40], and not resulting from “standardized measurement tools” [32].

The other 19 interventions conducted quantitative studies, although only five were randomized controlled trials (RCTs) [35, 36, 42, 50, 44] and one was a group randomized trial [33]. The remaining interventions all relied on standardized tools to measure loneliness or social isolation or some quantifiable variable such as the volume of incoming and outgoing interactions [36] or the size of the social network [47]. Seven studies reported no significant differences, but the majority reported positive outcomes such as decreased loneliness (*n* = 9) and increased network size (*n* = 2; see Table 1, Conclusion).

All but two interventions had measured participants' conditions at baseline, indicating that being lonely or isolated was a requirement for inclusion but without reporting how this condition was determined [41, 43]. Two studies included participants that perceived themselves as lonely [30, 32], while most interventions measured baseline conditions with some form or variant of the UCLA (*n* = 13) or De Jong Gierveld (*n* = 4) loneliness scales.

Finally, we mention the lack of agreement on the effectiveness of video chat and social networks. Széman [41] reported that Skype (video chat) helped to strengthen family ties and expand interpersonal connections, as well as to encourage learning on how to use other tools such as e-mail and chat. However, a computer training intervention by Blažun et al. [32] found that levels of loneliness for those who used Skype did not change, while those less lonely after the training used mainly e-mail, not Skype. Also, regarding social networking sites, Ballantyne et al. [30] reported a decrease in loneliness as a result of using a social network for older adults. These sites gave more control for users to manage their loneliness by giving access to contacts at any time and with no need to leave home. On the other hand, Széman [41] noted that Skype was preferred to Facebook because it was simpler to use even after participants had become familiar with Facebook and its functionalities. It is interesting to note that all the aforementioned interventions were supported by desktop/laptop computers and offered off-the-shelf solutions. No other conflicting results were found.

### 3.2. Challenges and Intervention Strategies

With respect to our first research question, we found that the *lack of social relationships* and *infrequent contacts* have been the most commonly addressed challenges to overcome loneliness and social isolation. One prominent strategy has been to train older adults to use computers and Internet (*n* = 16) [30–35, 37–39, 41, 42, 46, 50, 51, 53], although few technologies built specifically for older adults implemented this strategy [35, 46, 54]. After an initial training period, older adults in these studies were left to put in practice the skills learned to get in touch with others, with the exception of a single intervention where participants received continuous training [39]. Another strategy has been to ensure that the participants had someone to interact with (*n* = 10). These would usually be health professionals responding or contacting participants with some frequency for checks or to facilitate the intervention [37, 46, 47, 54] or other participants such as relatives who agreed to contact and support older adults during the study period [36, 48]. Other interventions had volunteers making phone calls [43], trained interviewers having video chat conversations [44], and trained helpers linked to a virtual companion [40]. One intervention ensured interactions by design, requesting participants to post and comment on Facebook daily [51].

*Usability and acceptance* of the technology by older adults were also challenges taken into account. Here, strategies have been to employ familiar devices, such as the telephone [43] or television [49]; devices that researchers regarded as more accessible and friendly, such as tablets [36, 40, 46, 48, 52]; or devices that researchers considered simple enough to require minimal [35, 47] to no training, such as touch-screen computers [44, 45]. Finally, some interventions specifically aimed at solving *conversational problems*, such as the lack of conversation topics. One strategy was to provide context with educational content in virtual groups or classrooms (e.g., about health), which allowed participants to discuss and share personal experiences [35, 45, 47, 49, 54]. An intervention reported that the shared experience provided topics of conversation for the less active participants [45]. Other interventions prepared conversation around topics such as the childhood and hobbies of participants [44] or implemented *buddy* systems based on common interests [35]. Although not designed as a part of the intervention, participants who received weekly phone calls [43] also reported shared interests as a way to break the ice as well as to establish a “meaningful reciprocal relationship” and mentioned the importance of knowing about others' lives and events and wanting to talk about ordinary, everyday topics. Pictures as prompt for conversation was also common, retrieving relatives' pictures from social media [36], using pictures during videochats [44], or enabling sharing with family and friends [33, 48, 49, 52].

### 3.3. Technology Supporting Interventions

In answer to our second research question, we found that Internet access was fundamental to support long-distance interactions in all interventions, except for the telephone befriending service [43]. On top of Internet, different combinations of technologies were incorporated, including general Internet use for interaction (e.g., discussions in forums) and e-mail (*n* = 10; see Table 2, Technology), video chat (*n* = 10), social networks (*n* = 8), virtual spaces or classrooms with messaging capabilities (*n* = 5), messaging services (*n* = 3), virtual companions (*n* = 1), and phone calls (*n* = 1).

Off-the-shelf solutions were favored (*n* = 15; see Table 2), including the social networks Facebook and About my age (a social network for older adults), Skype (video chat), WhatsApp, and Line (messaging services), a landline telephone service, as well as standard applications to use e-mail and Internet. Tailored solutions, designed specifically for interventions (*n* = 10) include systems that facilitated virtual spaces and messaging capabilities [35, 45, 47, 49, 54], systems that enabled different interaction channels via simplified interfaces [36, 48, 52], a custom video chat system that allowed calling by simply touching the screen [44], and a virtual companion controlled remotely by a trained helper [40].

Computers, along with the mouse and keyboard as input devices, were preferred for supporting interventions (*n* = 10; see Table 2), closely followed by tablets (*n* = 9) which seem to be most popular among more recent studies. Other interventions employed mobile phones (*n* = 3), traditional telephones (*n* = 1), customized TV sets (*n* = 1), and touch-screen computers (*n* = 3), one of which had a telephone handset attached to the screen so that users could get calls as they would on a regular telephone [45].

While interventions clearly report on the technology and devices used, features and channels used for communication are less discussed. This information is useful to gain insight into participants' preferences and adoption, and it is usually reported in terms of “most used” features. Some interventions report that comments and likes are preferred over predetermined messages [54]; the latter are usually not used much [46, 48] although reported to be useful when starting to use systems [48]. Other interventions [49, 53] indicate that interaction features are used as much as content consumption features (e.g., messaging and reading news). Studies with more qualitative insights praised video chat for allowing users to see people on the other side, which was particularly important with grandchildren [41]. One intervention even delivered fully remote training using Skype [39]. Interventions also reported that participants used Internet and computers to reengage in old interests, explore content, or participate in online communities [30–32, 39, 41], sometimes achieving a notorious improvement, even allowing to overcome depression [41].

In terms of understanding the human factors in the interventions and the relation between users and technology, we found that all interventions required older adults to use the technology on their own, although some studies reported that assistance was necessary for a long period [48, 52]. Aside for the telephone befriending service, all provided some kind of training or support (see Table 2, Training or support). Nonetheless, some interventions did require participants not to be proficient with technology on which they would receive training, for instance, having none to limited experience with social networks [51] or no computer experience [30–32, 39, 41]. Interestingly, two tablet-based interventions reported requiring no computer experience [36, 47]. The remaining interventions did not required lack of experience to participate.

With respect to the difficulties in interacting with technology, White et al. [42] reported on computer users having problems with vision, colors on the screen, the mouse, and remembering how to use e-mail and Internet. Other computer users withdrew from their studies because learning how to use the computer was too difficult [30, 31] or they had found a better alternative [31]. In touch-screen computer interventions, participants reported difficulties to join into group conversations and limited privacy settings and amount of characters per message, as well as frustration on the disengagement of others [45]. The lack of participation of others is echoed by more recent studies, mentioning that relatives did not respond often due to using WhatsApp (not supported in the study) more than e-mail [52] and that not receiving responses could increase the perception of loneliness [48].

Tablet users reported feeling silly talking to a virtual pet, problems with audio, and delay in messages [40]. A number of studies also reported that reduced dexterity could lead to difficulties with texting and typing [33], with gestures such as tapping and swiping [48, 52], and coordination issues such as requiring to hold voice icon to record audio messages [37].

Usability, although not formally an outcome, was analyzed by some interventions. In a computer training course by Blažun et al. [32], which included e-mail, Internet, and Skype, participants self-reported on satisfaction (64% were very satisfied or satisfied) and ease of use (74% reported it was easy). On the other hand, some interventions using computers [32, 41] and tablets [40] reported initial feelings of uncertainty and fear regarding use and adoption of technology. These interventions also reported that such feelings were overcome in time, as participants gained confidence and familiarity, thanks to both training and use. One computer-supported study indicates that 80% of participants reported that it was easy to become skilled at using the system. Studies with touch-screen computers [50] and tablets [48, 52] also reported increased confidence. Tablet studies, in particular, reported positive results with ease of use of the system [53] or interface [48] albeit some adaptation time required for gestures [52].

### 3.4. Social Interaction and Contacts

In relation to our third research question, out of the 25 interventions, 20 involved online groups (see Table 3), while in the remaining five, participants could only contact one other person (one-to-one interventions).

The majority of interventions (*n* = 19; see Table 3, Contacts) focused on interactions between older adults and their family and friends. Eight interventions had explicitly planned for contact with family and friends: Gutierrez et al. [36], Garattini et al. [45], and Neves et al. [48, 52] asked participants to choose relatives and friends to be added to their contact list, while Larsson et al. [39] had “searching for relatives” as a task on their program. The other three taught participants to communicate with family and friends via Internet [31, 34] or Skype [41].

Interestingly, Széman [41] reported that participants wanted to contact only their families in the beginning, but later asked to expand their network to include old friends, acquaintances, new people, and finally other participants. Initial apprehension to contact new people is also reported by [47]. Eleven interventions reported interactions between older adult study participants, with one even designed to “encourage social interaction among strangers” [45]. Among studies comparing frequency of contact, Fokkema and Knipscheer [31] reported that, out of 12 participants, ten had contact with family and acquaintances and three with other study participants. Baez et al. [46] report that group messages between participants was used more than one-to-one private messages, suggesting that more time might be needed before developing meaningful relationships.

One-to-one interventions limited contacts to family, friends, and acquaintances [48, 52], as well as trained interviewers [44], trained helpers [40], or volunteers [43], put in place for the interventions. However, interventions that included general Internet use and social networks allowed participants to meet new people. Larsson et al. [39] even had “finding a new friend with the same interests” as a program goal and contacting an unknown person through Internet as a task. Authors who replied to our inquiry on how new people were met said that most new contacts were other participants reached through forums [31] or that participants met others in social network pages about shared interests and during online activities offered by the site, such as “quiz night online” [30].

Intergenerational relationships were also indicated as important. Three computer training interventions explicitly mention interactions with young people. Blažun et al. [32] reported benefits for both parties, with younger volunteers teaching elders new ICT skills, and at the same time, learning by themselves from the life stories of the older adults. White et al. [42] reported that “some participants agreed to be e-mail pals with middle school students”, and Széman [41] reported that opportunities to contact grandchildren was the “biggest motivation” for participants. Studies by Barbosa et al. [48, 52] also report that some participants were more engaged with their grandchildren, communicating with them especially, and being happy about having the chance to see them grow through video.

Interactions in person also occurred. Sometimes, participants met during the interventions and formed groups: a computer interest group [42] (which got to publish a newsletter for the community), group workshops [53] and training [37], and discussion and support groups [30], for example, to watch and discuss YouTube videos [33]. Three interventions included support or teaching imparted in person by relatives or volunteers [32, 36, 41]. Sometimes, participants also wanted to meet in person as a result of the interventions. Myhre et al. [51] reported that participants used Facebook to arrange face-to-face meetings, after forming and maintaining relationship during the study period. Banbury et al. [47] also reported that six participants met after talking via videoconference.

In addition, visits were made in order to provide assistance or to make sure that systems were working properly (see Table 3, Contact with research staff). Especially, in interventions that provided education for computer and Internet use, visits were more or less frequent after the training period [30–32, 34, 39, 41, 42]. Few interventions did not report visits to participants: one-to-one interventions including video chat with the trained interviewer [44] (one visit for setup only) and the telephone befriending service [43], as well as studies that relied on the family to support the older adults [36] or reported offering remote support throughout [54]. Since our focus is on loneliness and isolation, it is worth noting that, in a large part of the interventions, there were visits to participants, which may have had an effect on the results.

## 4. Discussion

In the following, we analyze the findings from our research questions. In terms of challenges addressed by interventions, the strategies applied, and the intervention outcomes (RQ1), we observed that most interventions have dealt with the lack of social relationships and infrequent contacts by training participants in the use of computers and Internet. While results have been positive, and it is true that training participants or providing simple technology might solve the digital divide, such strategies do not guarantee access to contacts or frequent interactions. We argue that it is important to address the barriers directly, targeting challenges with technology that incorporates strategies by design. Interventions providing simple technology also ensured interaction as a strategy, and most of these interventions have reported positive outcomes. More studies taking this strategy would contribute evidence allowing for comparison with studies that train participants. Also, some studies have tried to improve conversations, by providing some contextual information and conversation topics. This area seems promising, especially in light of the surge of artificial intelligence and conversational agents. As fully automated conversational agents were successful on interventions for young adults with symptoms of depression and anxiety [55], such agents could be designed to target loneliness and social isolation and adapted to help guide conversations and provide conversational cues.

We also note that future studies should look into providing stronger evidence on the impact of the interventions conducted. Despite a majority of interventions reporting positive outcomes, and relying on quantitative methods, only five were RCTs. Studies disagree on the effectiveness of the technologies used (e.g., video chat and social networks), and some qualitative studies reports were obtained without standard measurement tools [32] or based on perceptions [40], making results hard to interpret and analyze. These findings are in line with previous reviews which have already highlighted weak methodologies [24] and noted that the quality of studies does not allow establishing conclusive remarks on effectiveness [25].

While we are advocating for more rigorous methodologies, we are not suggesting that qualitative outcomes should be abandoned in favor of quantitative ones. Qualitative studies are indeed useful to provide insight, especially on the motives behind behaviors. In this review, we have found studies reporting on the reasons for technologies not being adopted or reasons for people to interact more (or less) with others, uncovered through qualitative methods. In this sense, a methodological guide to designing studies in this area would greatly benefit researchers, especially those from the IT field, who might be less familiar with user studies with vulnerable subjects.

With respect to the technology used in interventions and how this technology was used by older adults (RQ2), we found that desktop/laptops make for a big part of the devices used to support interventions. Considering how commonplace long-distance interactions are nowadays and the availability of devices (e.g., mobile phones), we were surprised to find that studies based on desktop computers were so common, especially, since age-related limitations experienced by older adult computer users [56] might hinder the interactions enabled, thus leading to poor intervention outcomes. However, we have spotted a trend in recent studies to favor tablets, and there are a few studies based on mobile and smart phones.

We also found less solutions designed specifically for older adults than studies relying on off-the-shelf technologies. This might be due to the higher investment that designing tailored technologies requires. Nonetheless, previous research shows that tailored tools could increase adoption [57] while more general solutions (e.g., Facebook) could pose challenges for older adults [58] and present asymmetries in the interactions (especially for intergenerational communications [59]). This presents a great opportunity for human computer interaction researchers to collaborate with technology-supported interventions to facilitate long-distance interactions for older adults.

The prevailing technologies were e-mail and general Internet use for interaction (e.g., discussions in forums), closely followed by social networks and video chat. We must note, however, that while we have identified some studies using more recent technologies such as messaging services (e.g., WhatsApp) that allow for picture sharing and reaching relatives more conveniently, we still observe a disconnect between the latest technologies available and those used to conduct formal studies.

Many interventions enabled a combination of features and channels for interaction; however, few reported on how and how frequently these were used. Since such reports are scant, we cannot assess technology adoption or effectiveness. Therefore, we recommend future interventions to add formal reports on usability (e.g., the System Usability Scale [60]) and to quantify features and interaction channels used by participants. The adoption of technology by older adults largely depends on learnability and perceived difficulty of use [61]. Using standard instruments to measure usability is key to explain the success of technology-supported interventions, while failing to address usability might raise concerns about the validity of the intervention.

Finally, with respect to the social interactions enabled (RQ3), we found that most interventions enabled interaction with online groups, rather than with one person put in place specifically for the intervention. Family and friends were the contact group reported by a majority of the interventions, some highlighting intergenerational relationships as particularly important for older adults. Nonetheless, here, we also lack quantitative information on the frequency of contact. Since all interventions with online groups included at least two different groups of people (e.g., family and friends and other participants), we cannot tell whether older adults prefer to contact certain groups nor assess the impact the type of relationship has on the effectiveness of interventions. Friendship relationships, for instance, have been associated with stronger effects for subjective wellbeing [62] as compared with familial relationships.

The need for quantitative information also applies to the channels used to interact with people from particular groups. For example, Széman [41] reports that older adults enjoyed seeing their grandchildren through video chat. Future studies should consider analyzing the impact on effectiveness of the contacts enabled and the channels used for interactions, as well as quantifying interactions and the contacts reached. This would allow to better understand the motivations and opportunities that exist for conversation between older adults and others.

Furthermore, despite assessing the effect of long-distance interactions, many studies reported interactions in person during the intervention (e.g., with other participants, with researchers). If interactions were frequent, the effect on intervention outcomes should be considered.

## 5. Conclusions

For interventions, technology had the fundamental role of enabling long-distance interactions and was used for support in different ways. By facilitating more channels for interaction and providing access to larger audiences, it allowed participants to expand social networks, strengthen existing ties, providing social support, or build community rapport. However, since existing interventions are few, they tell us about the feasibility of using technology for long-distance interactions, but it is still unclear how technology is actually used, what limitations and opportunities exist, and how these affect the success of the intervention.

Therefore, we highlight some recommendations for researchers approaching this field of study. First, on the study methods, it is important to (1) design studies as RCTs, (2) leverage standard instruments for measuring loneliness and social isolation, and (3) consider the potential impact of continued (and in person) contact with participants on measurements. This may seem obvious, but we found few studies with these characteristics. We also recommend to report and discuss separately the results for each interaction channel and by the type of relationship (e.g., with friends, children, and grandchildren), since without this information, it is hard to infer what worked. Second, in terms of challenges, open opportunities lie in studying how technologies can facilitate and improve conversation (e.g., by presenting shared interests as topics), as opposed to enabling them. Finally, a vast majority of current research has focused on training for using a specific technology. To date, little attention has been paid to (1) designing interventions that enable or encourage usage of technology in specific ways (e.g., organizing and encouraging access to chat rooms with specific topics) and on (2) using persuasive technologies that introduce motivational elements and help users initiate and sustain conversations on shared interests. We feel that addressing these gaps in current research can lead to a better understanding of the role technology can play in tackling loneliness, helping to alleviate one of the modern ailments of our society.

## Figures and Tables

**Table 1:** Summary of the interventions analyzed.

Intervention	Technology/device	Study^a^: length/participants/age	Strategies	Study settings/methods	Outcomes: primary/secondary	Measurements^b^/Conclusion
Ballantyne et al. [30]	Social network (About my age)/computer	3 months/4/69–85	Internet and computer training	Home/pilot study, prepost interviews	Loneliness	Interview/decreased loneliness

Blažun et al. [32]	E-mail, Internet, and Skype/computer	3 weeks/45/66 in Finland, 77 in Slovenia	Internet and computer training	Home in Finland, residence in Slovenia/prepost test no control; no standard tool for assessment	Loneliness, ICT knowledge, and experience	Questionnaire/decreased loneliness

Cattan et al. [43]	Phone calls/telephone	>3 months/34/55–95	Familiar, simple technology; ensure interactions	Home/mixed methods	Health and wellbeing/loneliness	Questionnaire and interview/decreased feelings of loneliness, increased socialization

Cotten et al. [34]	E-mail, Internet, and Facebook/computer	1–2 weeks/205 (79; 126)/82.8	Internet and computer training	Facility/cross sectional analysis	Loneliness and social isolation/quantity and quality of communications	Hughes 3-items LS (UCLA-based) and questionnaire/decreased loneliness, not social isolation

Dodge et al. [44]	Video chat/touch-screen computer	6 weeks/83 (41; 42)/80.5	Familiar, simple technology; ensure interactions; provide conversation topics	Facility/randomized controlled trial	Cognitive function/loneliness	Hughes 3-items LS (UCLA-based)/no difference

Fokkema and Knipscheer [31]	E-mail and Internet/computer	3 years/26 (12; 14)/66 in intervention, 68 in control	Internet and computer training	Home/interrupted time series, nonequivalent control group prepost test	Loneliness	DeJong 11-items LS and questionnaire/decreased loneliness

Garattini et al. [45]	Broadcast, messages, and calls/touch-screen computer-phone hybrid	10 weeks/19/65–84	Familiar, simple technology; provide conversation topics	Home/mixed methods, exploratory study	Feasibility/social connectedness	DeJong 6-items LS, log, interview, questionnaire/helped social connection and created interactions

Larsson et al. [39]	E-mail, Internet, Skype, and Facebook/computer	3 months/30/61–89	Internet and computer training	Home/randomized crossover study	Loneliness/satisfaction with social contacts online and offline	UCLA LS/decreased loneliness (significant in both groups); satisfaction with social contacts inconclusive

Machesney et al. [40]	Virtual companion/tablet	1 week/13/65–93	Familiar, simple technology; ensure interactions	Home/one group observational study	Loneliness	UCLA LS/decreased loneliness

Széman et al. [41]	E-mail, Internet, Skype, and Facebook/computer	>6 months/15 (program), 25 (pilot)/>75	Internet and computer training	Home/case study	Loneliness	Observation/increased size of social network

White et al. [42]	E-mail, Internet/computer	5 months/93 (48; 45)/71 in interventions, 72 in control	Internet and computer training	Facility/randomized controlled trial	Loneliness	UCLA LS (modified anchors)/decreased loneliness (nonstatistically significant)

Baez et al. [46]	Virtual classroom, messages, and predefined messages/tablet	10 weeks/40 (20; 20)/71.5	Internet and computer training; familiar, simple technology; ensure interactions	Home/randomized pilot trial	Training adherence/loneliness and social wellbeing	Hughes 3-items LS (UCLA-based)/no significant difference

Czaja et al. [35]	E-mail, Internet, virtual classroom, and messages/computer	12 months/300 (150; 150)/76.15	Internet and computer training; familiar, simple technology; provide conversation topics	Home/randomized controlled trial	Loneliness and social isolation/attitude towards technology and proficiency	Hawthorne friendship scale, Cohen perceived social support scale, Lubben social network size, UCLA LS v3/decreased loneliness and social isolation

Banbury et al. [47]	Skype/tablet	44 weeks/52/73.0	Familiar, simple technology; ensure interactions; provide conversation topics	Home/nonrandomized noncontrolled prepost test	Educational goals/social support	Social (egocentric) network analysis interviews, focus groups/increased network size

Barbosa et al. [48]	Messages (video, photos, audio, predefined)/tablet	3 months/12/82.5	Familiar, simple technology; ensure interactions	Facility/feasibility study	Feasibility/social connectedness	Hughes 3-items LS (UCLA-based), Abbrev. Duke social support index/increased social interactions, high perceived social connectedness

Chiu and Wu [33]	Line (messaging service), YouTube/tablet	5 months/54 (19; 18; 17)/73.0	Internet and computer training; provide conversation topics	Facility/group randomized trial	Cognitive, physical functioning and psychological wellbeing/quality of life	CES-D Chinese version, Taiwanese inventory of social supportive behavior/increased social support and satisfaction with contacts

Gutierrez et al. [36]	Video chat, messages, and photos/tablet	9 weeks/9/69–81	Familiar, simple technology; ensure interactions	Home/empirical in-the-wild study	Frequency of social interactions	One-way repeated ANOVA/increased social interactions

Isaacson et al. [49]	Virtual classroom, video chat, photos/TV, remote, and webcam	4-5 weeks/40/85.86	Familiar, simple technology; ensure interactions	Home/pilot study	Technology adoption/emotional wellbeing	UCLA LS v3, Lubben social network scale/decreased loneliness, increased social wellbeing and social network size

Jarvis et al. [37]	WhatsApp (messaging service)/mobile phone (smartphone)	3 months/29 (13; 16)/74.93	Internet and computer training; ensure interactions	Facility/randomized controlled study	Loneliness and social cognition/use of technology	YSQ short form, DeJong 6-items LS/decreased loneliness

Jarvis et al. [38]	WhatsApp (messaging service)/mobile phone (smart phone)	3 months/32 (15; 17)/70.42	Internet and computer training	Facility/experimental randomized comparative study	Loneliness	DeJong 6-items LS, focus groups/decreased loneliness

Morton et al. [50]	E-mail, Internet, Facebook, and Skype	4 months/76 (44; 32)/80.71	Internet and computer training	Some at home, some at facility/randomized 2 × 2 × 2 study	Cognitive and mental health/social network activity and satisfaction, loneliness	Social network activity index, UCLA LS v3/no difference in loneliness, increased social network activity

Myhre et al. [51]	Facebook	8 weeks/41 (14; 13; 14)/80.0 Facebook, 73.38 online diary, 79.29 Waiting list	Internet and computer training; ensure interactions;	Some at home, some at facility/3-arm study	Neuropsychological tests/social engagement	UCLA LS v3, MOS social support survey, Lubben social network 18-i scale/no significant difference

Barbosa et al. [52]	Video chat, photos, audio recording, predefined messages	2 months/5/87.2	Familiar, simple technology	Facility/embedded case study	Feasibility and adoption/social connectedness	Hughes 3-items LS (UCLA-based), Abbrev. Duke social support index/no significant difference

Pauly et al. [53]	E-mail, Internet, social network, and messages	>6 months/92/67.7	Internet and computer training	Home/prepost, repeated measures study	Physical activity/loneliness and executive functioning	Self-reported questionnaires, R-UCLA LS/no significant difference

Tomasino et al. [54]	Virtual classroom and messages	8 weeks/47/69.6	Internet and computer training; ensure interactions; provide conversation topics	Home/pilot study	Depression, tech use and usability/social support and isolation	PROMIS social isolation 6-i, social Provisions scale/no significant difference

^a^The number of participants in controlled studies is shown in parentheses (intervention; control); participants' age is indicated as mean, age range, or as reported in the study. ^b^LS stands for Loneliness Scale.

**Table 2:** Technology used in interventions.

Intervention	Technology	Custom or off-the-shelf	Devices	Technology ownership/experience	Training or support
Ballantyne et al. [30]	Social network (About my age)	Off-the-shelf	Computer	Nonproficiency required	Initial training sessions
Blažun et al. [32]	E-mail, Internet, and Skype	Off-the-shelf	Computer	Nonproficiency required	Initial training sessions
Cattan et al. [43]	Phone calls	Off-the-shelf	Telephone	N/A	N/A
Cotten et al. [34]	E-mail, Internet, and Facebook	Off-the-shelf	Computer	Did not report	Initial training sessions
Dodge et al. [44]	Video chat	Custom	Touch-screen computer	No previous use of PC (15%)	Visits for setup; no training
Fokkema and Knipscheer [31]	E-mail and Internet	Off-the-shelf	Computer	Nonproficiency required	Initial training sessions
Garattini et al. [45]	Virtual room, calls, messages, and broadcasts	Custom	Touch-screen computer with phone handset	No computer ownership (68%)	Visits for training and support
Larsson et al. [39]	E-mail, Internet, Facebook, and Skype	Off-the-shelf	Computer	Computer ownership and nonproficiency required	Visits for training and support; remote training
Machesney et al. [40]	Virtual companion (pet avatar)	Custom	Tablet	Did not report	Continuous visits; remote support
Széman et al. [41]	E-mail, Internet, Facebook, and Skype	Off-the-shelf	Computer	Nonproficiency required	Initial training sessions
White et al. [42]	E-mail and Internet	Off-the-shelf	Computer	Owned a PC (9%); no previous experience (60%)	Continuous visits; remote support
Baez et al. [46]	Virtual classroom, messages, and predefined messages	Custom	Tablet	Did not report	Initial training; remote support
Czaja et al. [35]	E-mail, Internet, virtual classroom, and messages	Custom	Computer	Participants had minimal computer or Internet use experience	Initial training; check visits; remote support
Banbury et al. [47]	Skype	Off-the-shelf	Tablet	Most had no previous video conference experience	Visit for setup
Barbosa et al. [48]	Messages (video, photos, audio, and predefined)	Custom	Tablet	Moderate (5), basic (3), or no experience (4)	Initial training; weekly visits for support
Chiu and Wu [33]	Line (messaging service) and YouTube	Off-the-shelf	Tablet	No computer learning experience (82%)	Training sessions (long period)
Gutierrez et al. [36]	Video chat, messages, and photos	Custom	Tablet	First-time as computer user required	Provided by family member
Isaacson et al. [49]	Virtual classroom, video chat, and photos	Custom	TV, remote, and webcam	Many not proficient with smart phones/computers	Visit for setup and training
Jarvis et al. [37]	WhatsApp (messaging service)	Off-the-shelf	Mobile phone (smart phone)	Used mobile to contact family and friends (55%), none had used WhatsApp	Initial training; weekly visits for support
Jarvis et al. [38]	WhatsApp (messaging service)	Off-the-shelf	Mobile phone (smart phone)	N/A	Initial training; weekly visits for support
Morton et al. [50]	E-mail, Internet, Facebook, and Skype	Off-the-shelf	Touch-screen computer	Required no current access to Internet	Continuous visits; remote support
Myhre et al. [51]	Facebook	Off-the-shelf	Computer and tablet	No social network or minimal use required, tablet/computer ownership required	Initial training
Barbosa et al. [52]	Video chat, photos, audio recording, and predefined messages	Custom	Tablet	All participants inexperienced with tech, save one	Initial training; weekly visits for support
Pauly et al. [53]	E-mail, Internet, social network, and messages	Off-the-shelf	Tablet	None or very little experience with portable electronic devices (67%)	Initial training; workshop during intervention
Tomasino et al. [54]	Virtual classroom, messages	Custom	Computer, tablet, mobile phones	Required Internet access and basic Internet skills	Remote support

**Table 3:** Social interactions and contacts.

Intervention	Online group or one-to-one	Contacts	Contact with research staff
Ballantyne et al. [30]	Online group	Family and friends, new people	Weekly visits first, then fewer; phone calls at most 1 h/week
Blažun et al. [32]	Online group	Family and friends, new people	Training once a week; 4 h in Finland and 3 h in Slovenia
Cattan et al. [43]	One-to-one	Volunteers (predefined)	Variable number of weekly calls
Cotten et al. [34]	Online group	Family and friends, new people	Eight-week training (data from first 2 weeks)
Dodge et al. [44]	One-to-one	Predefined (trained interviewers)	Video chat 30–35 min/day; 5 days/week
Fokkema and Knipscheer [41]	Online group	Family and friends, new people, other participants, acquaintances	5 × 2 h lessons; visits every 2–3 weeks
Garattini et al. [45]	Online group	Family and friends, other participants	4 × 1 h visits; messages via app; weekly calls (extra calls for technical issues)
Larsson et al. [39]	Online group	Family and friends, new people, and other participants	Individual meeting offered weekly, group meeting every 2 weeks
Machesney et al. [40]	One-to-one	Predefined (trained helpers)	Visits and phone calls, available 24/7
Széman et al. [41]	Online group	Family and friends, new people, and acquaintances	1 × 1.5 h lesson; 1 h visits twice a week
White et al. [42]	Online group	Family and friends, new people	3 × 2 h lessons, three 1 h lessons; trainer visits 2 h/week
Baez et al. [46]	Online group	Other participants, predefined (coach)	1.5 h training model before baseline; support messages/calls
Czaja et al. [35]	Online group	Family and friends, other participants	Initial setup, 3x check visits, calls at week 1, months 3 and 9
Banbury et al. [47]	Online group	Family and friends, predefined (facilitator), and other participants	Minimal training
Barbosa et al. [48]	One-to-one	Family and friends, acquaintances	Individual training at before deployment; weekly support visits
Chiu and Wu [33]	Online group	Family and friends	90-min ICT training sessions weekly for 12 weeks
Gutierrez et al. [36]	Online group	Family and friends	Unaccounted frequency, support provided by family member
Isaacson et al. [49]	Online group	Family and friends, other participants	Visit for setup and training and after 4–5 weeks
Jarvis et al. [37]	Online group	Family and friends, predefined (facilitator)	8x 90-min training session (during first 4 weeks), then weekly support visits
Jarvis et al. [38]	Online group	Family and friends, other participants	2x 90-min training session/week over 15 days, then weekly support visits
Morton et al. [50]	Online group	Family and friends, other participants, and acquaintances	3x 90-min training/week (month 1); session every 2 weeks, 1 h remote support alternate weeks (month 2), then 1 month of remote support and no visits
Myhre et al. [51]	Online group	Other participants	3 × 2 h training session for a week
Barbosa et al. [52]	One-to-one	Family and friends	1x individual training session, then weekly visits
Pauly et al. [53]	Online group	Family and friends	2x training sessions before intervention, 3 h customized workshop during intervention
Tomasino et al. [54]	Online group	Other participants, predefined (coach)	Remote support throughout the study
